# Antimicrobial agents – optimising the ecological balance

**DOI:** 10.1186/s12916-016-0661-z

**Published:** 2016-08-05

**Authors:** Sze-Ann Woon, Dale Fisher

**Affiliations:** 1 Division of Infectious Diseases, University Medicine Cluster, National University Hospital, Singapore, Singapore; 2 Department of Medicine, Yong Loo Lin School of Medicine, National University of Singapore, Singapore, Singapore; 3 National University Health System, NUHS Tower Block, 1E Kent Ridge Road, Level 10, Singapore, 119228 Singapore

**Keywords:** Antibiotic stewardship, Antibiotic policy, Antibiotic pipeline, Antimicrobials, Antibiotics

## Abstract

**Background:**

There is no more challenging a group of pharmaceuticals than antimicrobials. With the antibiotic era came great optimism as countless deaths were prevented from what were previously fatal conditions. Although antimicrobial resistance was quickly identified, the abundance of antibiotics entering the market helped cement attitudes of arrogance as the “battle against pestilence appeared won”. Opposite emotions soon followed as many heralded the return of the pre-antibiotic era, suggesting that the “antibiotic pipeline had dried up” and that our existing armament would soon be rendered worthless.

**Discussion:**

In reality, humans overrate their ecological importance. For millions of years there has been a balance between factors promoting bacterial survival and those disturbing it. The first half century of the “antibiotic era” was characterised by a cavalier attitude disturbing the natural balance; however, recent efforts have been made through several mechanisms to respond and re-strengthen the antimicrobial armament. Such mechanisms include a variety of incentives, educational efforts and negotiations. Today, there are many more “man-made” factors that will determine a new balance or state of ecological harmony.

**Conclusion:**

Antibiotics are not a panacea nor will they ever be inutile. New resistance mechanisms will be identified and new antibiotics will be discovered, but most importantly, we must optimise our application of these extraordinary “biological tools”; therein lays our greatest challenge – creating a society that understands and respects the determinants of the effectiveness of antibiotics.

## Background

The discovery of penicillin brought great hope for the treatment of infectious diseases. However, two decades later, in the late 1940s, acceptance, mass production and distribution led to the advent of the concept with which we are all now very familiar – antimicrobial resistance (AMR). As other classes of antibiotics emerged, a similar pattern ensued from discovery through increasing utilisation to resistance. Poor infection control practices in healthcare settings facilitated the transmission of resistant organisms, amplifying the effect of antimicrobial usage. The healthcare industry responded with two major programmes – Infection Control in the 1980s and Antimicrobial Stewardship in the last decade. These programs rely on the end user; however, while the importance of such global programmes cannot be disputed, these efforts alone are inadequate against the emergence of AMR.

If clinicians are to have an antimicrobial arsenal to treat infectious diseases, a combination of efforts to both preserve the value of what is currently available and to promote drug discovery is needed. Preservation of existing antimicrobials ultimately relies on minimising their use in all circumstances. In humans, there are many strategies to support this goal, including the implementation and adherence to guidelines provided by international organisations such as the World Health Organization (WHO) and the Centres for Disease Control and Prevention, by national bodies and individual institutions, as well as through the implementation of rapid (possibly point-of-care) diagnostic tests for AMR. Further, infection prevention activities also need to be implemented to minimise the need for treatment.

In addition to direct human ingestion, animal husbandry is a major setting for antibiotic consumption that allows indirect human exposure to the same antibiotic or the resistant organisms (or their resistance genes) emerging via animals farmed as a source of food. The strategies to curb this are considerably more complex, requiring engagement of a number of non-medical players less appreciative of the ramifications of antibiotic misuse.

Drug discovery has suffered from a lack of exploitable bacterial binding sites, leading to the slow emergence of truly novel agents. Modifying existing antimicrobial agents to achieve theoretical benefits is often seen as commercially safer. Many pharmaceutical companies have shifted towards treatment of chronic conditions in order to lessen the financial risk. Therefore, this movement needs to be opposed with financial incentives and better protection for future and more novel antimicrobial discoveries. The antibiotic pipeline needs to be restored, but whether such efforts will be successful remains to be seen.

Along with the excessive morbidity and mortality, AMR poses a major economic burden, which helps justify the costs of AMR response programmes. In the United States (US), mortality from antimicrobial-resistant infections is reported at 23,000 deaths annually, with costs estimated at US$ 26 billion [1]. In Europe, the annual hospital death rate is estimated at over 25,000, with costs totalling more than EUR 900 million [2].

Humankind has enjoyed the capacity to treat bacterial infections for over 75 years. In this commentary, we present a number of efforts intended to help maintain this capacity moving forwards. These efforts could be regarded as more of a “bundle”, where none will have significant benefit if implemented in isolation. AMR is a natural phenomenon – there are behaviours that stimulate it and responses that minimise it. The fear of “re-entering the pre-antibiotic era” is as unlikely as the eradication of antimicrobial-resistant organisms. We do, however, have some control over where on this spectrum the value of our antimicrobial armament will sit in the future. While the development of other classes of therapeutics relies on building on an existing knowledge base, in the world of antimicrobials, advances need to be made while simultaneously protecting the current therapeutic options from the very real threats to their effectiveness.

## Controlling AMR

### Implementation of antimicrobial stewardship programmes

Bacteria are excellent at evolving mechanisms to counter the effects of antibacterial agents and this has been the way since ancient times, long before humans and modern medicine. Much has been written on the antibiotic resistosome, which has evolved from environmental bacteria and competitive pressure from other unicellular organisms in conjunction with the ability to receive genetic material both vertically (from “parent” bacteria) and horizontally from other organisms. Confronted with many mechanisms evident against all antimicrobials, it was time for a renewed respect for our relationship between microorganisms, antimicrobials and resistance mechanisms and drivers. In the human administration of antibiotics today, this is called stewardship.

Although the concept of antibiotic stewardship was raised in the 1980s, it had no validation until a randomised controlled study demonstrated that antibiotic use could be significantly reduced without adverse outcomes [3, 4]. Since then, it has grown and evolved into a prospective, formalised, multidisciplinary programme. Active interventions, such as discontinuing redundant antibiotics, transitioning from parenteral to oral therapy, optimising dose regimens and de-escalating from broad-spectrum to pathogen-directed therapies, have been shown to reduce costs and toxicity and prevent the selection of resistant organisms [5–7]. While the emergence of antibiotic-resistant organisms requires at least some antibiotic exposure, the relationship between the extent of use and resistance is less clear. It is intuitive that reducing antibiotic use is key to countering this natural phenomenon; however, social factors, including government corruption, have been postulated as more valid [8]. It is likely that corruption is a surrogate for poor behaviour in terms of stewardship, infection control, public health and the necessary government controls to affect and monitor healthcare and agricultural systems driving the spread of resistant organisms.

This lack of clarity of the benefits of a solitary intervention, such as antibiotic stewardship programmes, should not deter us from the very likely benefit (and at a minimum, lack of harm) of it as part of a holistic multipronged response. Although efforts have been made internationally to encourage such programs, this is still primarily limited to acute care settings and tertiary centres where there are infectious disease physicians or microbiologist champions. However, to optimise future benefits, programmes must be effective in all healthcare settings, including outpatients, aged care, peripheral/rural hospitals, the community and, importantly, animal husbandry. Stewardship practices should not be limited to antimicrobials used for treatment, but should include those used for prophylaxis, which vary considerably and are not supported by evidence. The role of good stewardship can be undermined when attention is excessively directed to seemingly more tangible strategies such as reinforcing the pipeline of new antibiotics. A balanced approach is required.

### Non-prescription antibiotics

Non-prescription, over-the-counter antimicrobial access occurs worldwide, particularly in Asia and Southern Europe [9]. This is associated with frequent adverse events, masking of underlying infection and a contribution to the issue of increasing AMR as its use does not take into account a qualified clinical opinion and local susceptibility patterns. Although the contribution of non-prescription antibiotic use to the worldwide spread of resistance is not known, it has been speculated to play an important role in producing high levels of resistance in those communities. In Chile [10] and Korea [11], improved regulation of non-prescription antibiotics has been associated with improved resistance profiles. Not only can the consumed antibiotic be the wrong choice, but there is evidence that the use of expired [12], counterfeit [13, 14] and inappropriately low antimicrobial doses [15–17], which are common associations, contribute further to the risk of treatment failure and resistance. Self-medication is a potentially important alternative to formal consultations, particularly in low-resource settings; however, in reality, such access to medication without a prescription is usually a cultural and system issue, as doctors are often available. In most populations, this issue can be resolved, but not without controversy. The challenges in changing this practice lie not only in implementing legislation that will prevent the use of over-the-counter antibiotics but increased awareness through educational interventions and, most importantly, easy access to professional help and affordable drugs.

### Reducing antibiotic use in animals and agriculture

Agriculture and food animals are important sources of AMR as they are exposed to enormous quantities of antibiotics and serve as a reservoir of antibiotic-resistance genes. Antibiotics have been used in animal husbandry, veterinary medicine and agriculture to prevent, control and treat infections for over 60 years. Antibiotic use in growth promotion is, however, controversial. It was discovered that small sub-therapeutic doses of penicillin and tetracycline could enhance weight gain by about 15–20 %, although the underlying mechanism of this action remains unclear [18–21]. Sweden became the first country to ban antimicrobial use as growth promoters followed by Denmark and Germany. The implementation of a European Union-wide ban occurred in 2006 [22]. Partial restriction exists in countries such as Mexico, South Korea, Taiwan and Hong Kong; however, widespread use of antimicrobial growth promoters still occur in the majority of the world. Transmission of resistance occurs through the food chain, through contact with people working with animals, such as farmers, and through manure-contaminated environments. The recent report of plasmid-mediated colistin resistance genes found in slaughtered pigs, raw food and humans highlights the risk of transmission from animals to humans [23]. Efforts have been made by some countries, including the US, to phase out the use of antibiotics in animal feeds. Regulating and raising awareness and support amongst farmers, food producers and retailers have taken place; yet, more still needs to be done globally [24]. There are many possible options for supporting the concerns of the farming community while reducing their antimicrobial use. These include establishing standardised universal surveillance of antibiotic use and resistance, ensuring appropriate training and prudent use of antibiotics, expanding the range of vaccines available for veterinary use, using phage therapy (the neutralisation of foodborne pathogens in animals [25] and control of plant pathogens [26]), and using predatory bacteria in combating pathogenic bacteria [27, 28].

## Reviving the antibiotic pipeline

Antibiotic discovery was strong from the 1940s to 1960s, but slowed for some decades until the introduction of linezolid in 2000 and daptomycin in 2003 [29]. Most of the early antibiotics were discovered by screening soil-derived actinomycetes; yet, over time, this source became exhausted and no longer provided novel compounds or a further understanding of the mechanisms of action. Efforts have since been redirected to producing synthetic antibiotics by modification of existing drugs to produce active analogues, but this has led to limited benefits. The subsequent development of high-tech platforms based on genomics and combinational chemistry, such as high-throughput screening and rational drug design, were employed but failed to identify compounds with effective antibacterial activity [30]. The number of large multinational pharmaceutical companies actively developing antibiotics declined as a result of this lack of progress in the field along with the financial risks.

Drug discovery and development is conventionally driven by financial motivation. For the private industry, aligning to an antimicrobial programme has more risk than other pharmacological fields. Taking new drugs to market requires considerable testing for safety and efficacy in each indication sought. Once resistance ensues, the clinical, and therefore commercial, value of the product falls. In the medical profession, physicians try to limit a new antibiotic’s utilisation to maintain its clinical value; however, paradoxically, this affects sales, in effect creating another disincentive to drug discovery. When these new drugs are used in infectious disease management they have a short-term curative application as opposed to drugs used in the treatment of chronic diseases. It is no wonder that this “antibiotic pipeline” is weak and requires support from government and non-government sectors.

### Supporting the discovery and development of new antimicrobial drugs

**Fig. 1:**
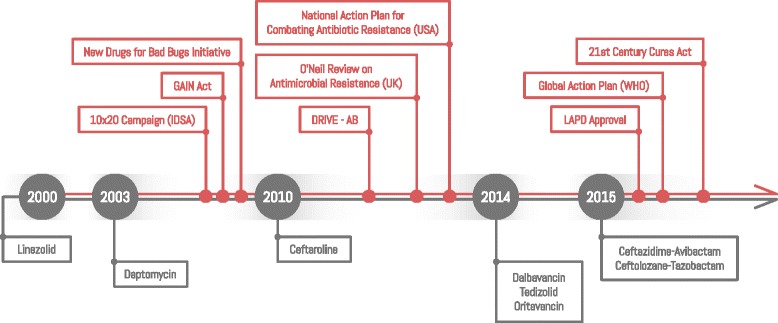
The antibiotics timeline since 2000. Since 2000, only a small number of antibiotics have been approved (below the timeline). Recently efforts have broadened to enhance the potential pipeline and also to protect the antibiotics currently available (above the timeline), by incentives and legislation

Recognising the waning value of the antimicrobial armament, in 2004, the Infectious Diseases Society of America proposed legislative, regulatory and funding solutions to counteract barriers in the development of new antibiotics [31]. These included tax incentives for development of priority antibiotics, measured liability protections, revising existing guidelines for clinical trials involving new antibiotics, and supporting public/private partnerships to increase funding. The so-called antibiotic pipeline could not flourish relying on conventional market forces alone, and the last decade or so has seen considerable attention to legislative support (Fig. 1). The extent of these efforts in supporting drug discovery was unprecedented.

The 10 × ‘20 campaign, launched by the Infectious Diseases Society of America in 2010, called for the development and approval of 10 novel antibiotics by 2020 [29]. The Global Action Plan by WHO aims to improve awareness of AMR, strengthen knowledge through surveillance and research, reduce the incidence of infection, optimise the use of antimicrobial agents and increase investment in new drugs, diagnostic tools and vaccines [32]. The O’Neil Review on Antimicrobial Resistance by Prime Minister David Cameron in the United Kingdom [33] and the release of the National Action Plan for Combating Antibiotic Resistant Bacteria by President Barack Obama [34], highlight the high level support for these efforts and augment the global response.

Many other initiatives and collaborations have recently been established to create new models for antibiotic development via incentive strategies. These include the provision of subsidies to lower antibiotic research costs for drugs with a large market, high potential sales and low risks. Sharing of scientific databases would facilitate collaboration between developers, minimising duplication and facilitating the dissemination of new information. Funding training and development of individuals specialising in research and development of antibiotics is key, as is financial support for small companies that are likely to produce marketable products but lack the capital to do so [35–37]. The European Commission’s Innovative Medicines Initiative [38] is a large public–private incentive programme supporting the rapid development of effective drugs through which the New Drugs for Bad Bugs (ND4BB) initiative [39] and, subsequently, the DRIVE-AB programme [40] were established with the aim of facilitating antibiotic discovery and development through various collaborations.

Another strategy was to compensate successful development as a way to motivate developers. The Generating Antibiotic Incentives Now Act was ratified in October 2012 by the US government and aimed to incentivize antibiotic discovery [41]. This included granting an additional 5 years of market exclusivity for antibiotics developed to treat serious infections, termed the qualified infectious disease product, and an additional 6 months for development of a companion rapid diagnostic test. This bill also entitled the new drug to priority review and fast-track approval, which in turn committed the Food and Drug Administration (FDA) to review its clinical trial guidelines and review processes. In addition, a streamlined clinical trial process for novel antibiotics, the Limited Population Antibacterial Drug approval, allows potential drugs to be studied via smaller, faster and less expensive trials [42]. Following this, a proposed US bill, the 21st Century Cures Act, offers qualified antibiotics Limited Population Antibacterial Drug approval and the option of transferable market exclusivity for up to 12 months, where the purchasers are required to donate a portion of the returns to AMR research and patient access programmes [43].

Perhaps a more well-rounded model is the WHO’s Global Consortium [44, 45], which provides support at the drug discovery stage via an open source platform as well as grants for academics. It aims to lower development costs and risk using patent buy-outs of successfully developed antibiotics (which facilitates antibiotic stewardship and negates the need for excessive marketing), funding of clinical trials and advance purchase commitments for generic distribution.

Additionally, many business models and incentive strategies have been proposed, with some placing emphasis on investment at the beginning of drug development while others reward only successful development of a drug [46]. Nevertheless, a successful package not only requires the creation of a supportive environment for investment that increases profitability and aligns both public and private priorities, but should also address public health objectives such as promoting antibiotic stewardship and improving patient access to new (potentially expensive) antibiotics.

### What’s new in the pipeline?

Since the approval of telavancin in 2009 and ceftaroline in 2010, five new antibiotics have been approved by the FDA, three of which have mainly gram-positive activity and two directed against gram-negative pathogens. Oritavancin and dalbavancin are long-acting, synthetic lipoglycopeptides with activity against methicillin-resistant *Staphylococcus aureus* and vancomycin-resistant *Enterococcus*, and are approved for treatment of acute bacterial skin and skin structure infections [47–49]. Tedizolid, an oxazolidinone-class antibiotic similar to linezolid, is licensed for treatment of these infections [50], with treatment for other indications underway, including hospital-acquired pneumonia. While these new drugs have the advantage of being utilised in the outpatient setting, thus avoiding prolonged intravenous use and length of hospital stay, they are still costly and questions remain as to their utility in the treatment of more serious clinical infections such as bacteraemia, endocarditis, osteomyelitis and device-related infections. Ceftolozane/tazobactam and ceftazidime/avibactam are approved for treatment of complicated intra-abdominal and urinary tract infections; nevertheless, the latter is only approved for use in patients with limited or no treatment options as it was the first drug to go through the recommended regulatory pathway and deemed to be an “unmet medical need drug” [51–53]. Both antibiotics have activity against extended spectrum beta-lactamases, AmpC producers and multi-drug resistant *Pseudomonas aeruginosa*, but only ceftazidime/avibactam has activity against Class A carbapenemases.

In May 2016, the Pew Charitable Trust, which maintains a regularly updated antibiotic pipeline, described 37 new antibiotics in development, of which 11 were in phase I clinical trials, 13 in phase II and 13 in phase III [54]. About a third of these have the potential to treat infections caused by *Enterococcus faecium*, *Staphylococcus aureus*, *Klebsiella pneumoniae*, *Acinetobacter baumannii*, *Pseudomonas aeruginosa* and *Enterobacter spp* (ESKAPE pathogens), and just over a third have activity against drug-resistant gonorrhoea, *Clostridium difficile* and carbapenem-resistant Enterobacteriaceae, which are pathogens considered an “urgent threat” by the Centres for Disease Control and Prevention [1]. Of drugs in phase III, only about 60 % will typically reach FDA approval level and only about half of these are active against gram-negative pathogens. Notably, none have promising activity against carbapenem-resistant *Acinetobacter sp.* or the Class D metallo-beta-lactamases (e.g. NDM-1) [54]. Of the 37 antibiotics, very few have a novel mechanism of action and none of these are yet in phase III studies. Nonetheless, there is reason for optimism in both the short and long term.

Bacteriophage treatment is a largely untested approach to antibacterial therapy and represents a revival of a previous option. These viruses infect and rapidly kill specific target bacteria. Nevertheless, none are in therapeutic practise although many are in development and at various stages of clinical trials [55]. Bacteriophages may prove to be novel antimicrobial agents with potential use in decolonisation or treatment of infection alone or in association with conventional antibiotics, and there is also specific potential for their use in infections complicated by biofilm formation. The side effect profile needs to stand the test of time, but the few studies to date suggest a good safety profile [56].

### A renewed hope for antibiotic discovery?

In January 2015, a group of scientists from Northeastern University, MA, described their discovery of an antibiotic, teixobactin, with a completely different chemical scaffold to that of existing antibiotics. It has gram-positive activity, which works through binding to precursors of bacterial cell wall polymers and appears to have no inducible resistance. This discovery involved a novel technology called iChip [57], a miniaturised, multichannel device that allowed antibiotic-producing soil bacteria to be cultivated and identified in their natural environment. Scouring the soil for new antimicrobial molecules has proven to be extremely challenging in the last decade as soil microorganisms cannot be grown under traditional laboratory conditions. This innovation can hopefully be applied to identify promising molecules with novel mechanisms of action.

## Improving affordability and improving patient access to antibiotics

When a new antibiotic reaches the end of the arduous pipeline and is registered, the traditional patent-based pharmaceutical model grants developers market exclusivity for a number of years, following which generic manufacturers are permitted development and marketing opportunities to the drug. During these early years with the patent in place, the pharmaceutical organisation has a window period to obtain financial returns for the research and development, clinical trials and notoriously expensive marketing, as well as shareholder profit; they control the market geographically and they control price. Most patients cannot benefit from drugs that are not distributed globally and financial returns are not usually found in low- and middle-income countries. In fact, most patients in high-income countries will struggle with meeting the costs as individuals; drugs not yet recognised in national pharmaceutical benefit systems or by insurers may lead to excessive costs for individuals even in these countries.

It is a “double-edged sword”, where we wish to improve access, yet not to the point where excessive use drives high levels of resistance. Any programme that improves access to individuals in need should build-in measures that minimise inappropriate use. The current model potentially creates incentive to encourage any consumption of the antibiotic, but only to those individuals and countries that can pay. In the last decade or so, several systems have provided remarkable contributions to access in low- and middle-income countries on a large scale, including the Global Fund (for malaria, tuberculosis and HIV), The Presidents Emergency Fund for AIDS Relief (PEPFAR) and the Global Alliance for Vaccines and Immunisation (GAVI). Ideally, similar efforts in the name of antibiotic access could ensue via negotiation and bulk purchasing or via subsidy. The crucial effort lies in delinking profits from sales volume. The scale of the antibiotic issue is different and the situation more complex, but some efforts have been initiated; examples include the Antibiotic Health Impact Fund, Antibiotics as Public Goods, and the Rewarding Antibiotic Development and Responsible Stewardship Programme, where antibiotics could be offered at a marginal cost and overall profitability of projects is tied to global health impact [46, 58]. Whilst still providing developers with a concrete return, it removes the motivation to oversupply the market, incentivises developers to distribute their new antibiotics, and facilitates access to those who need them most. Other strategies include patent buy-outs to successfully developed antibiotics and sales of a license for unlimited access to antibiotics at a small cost, hence allowing fair distribution, increased affordability and equitable access [58].

## Raising awareness and changing prescribing patterns and behaviour

There is no doubt that inappropriate antibiotic use is a significant contributor to the emergence of resistance [59, 60]. It has been suggested that 20–50 % of prescribing is unnecessary. In primary care, antibiotics are frequently prescribed for upper respiratory tract symptoms and the treatment of asymptomatic bacteriuria is associated with a higher prevalence of resistant organisms [61–64]. The prescription and use of antibiotics is influenced by a complex interplay of knowledge and attitudes of the prescriber and patient (and in the case of animal husbandry, the farmer). Patients who expect a prescription are more likely to get one [65, 66]. Many doctors would admit that patients’ expectations, peer pressure, fear of litigation, poor knowledge of microbiology and underutilisation of available guidelines play a role in driving poor antibiotic use [62, 67]. Few clinicians see the bigger picture, the antibiograms, the national epidemic curves, etc.; however, all prescribers and users are contributors to the evolving quantum of multidrug-resistant infections. There needs to be an increased awareness that the imprudent use of antibiotics does not usually exert its effect at an individual level, but to the society as a whole, with serious public health implications. A recent WHO assessment revealed that there is a lack of awareness among both the public and healthcare workers in this matter [68]. Several interventions to improve antibiotic use targeting both the public as well as healthcare providers, particularly multimodal campaigns, have shown positive outcomes in terms of adherence to guidelines and reduction of inappropriate prescription [69–71]. However, it remains unclear whether the effectiveness of these campaigns as well as the change in practice and behaviour are sustainable. Multifaceted mass media and educational campaigns targeting both the public and healthcare professionals, but culturally tailored to the particular community, should be held with active engagement of all levels of authority. This should be performed in a coordinated and comprehensive manner that addresses all aspects of behaviour such as capability, opportunity and motivation [72]; changing health behaviour is difficult and requires significant and sustained commitment and investment.

## Conclusion

**Fig. 2:**
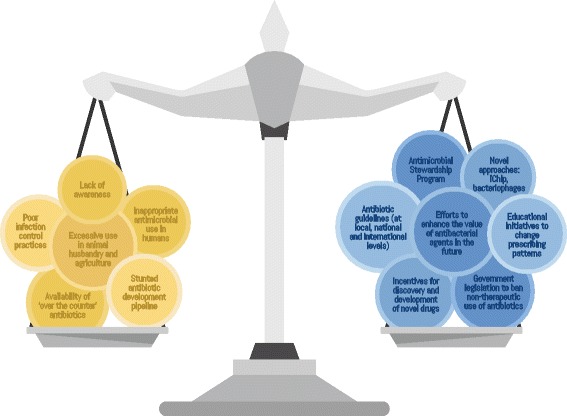
The balance that will determine the effectiveness of antimicrobials as a class. The value of the antimicrobial armament in the future is dependent on numerous opposing forces. Factors on the left of the scale represent those diminishing the value while those on the right aspire to counter these. The ultimate worth of antimicrobials in the future will be realised in how these opposing forces balance out

Antibiotics are unique therapeutics and a human resource. They are the only drug class that, when used in an individual, it can affect others. Their misuse has resulted in a shift in the ecological balance between resistant and susceptible strains in the flora of humans, animals and the environment, and there are several determinants of where the balance will settle (Fig. 2). As healthcare providers, we hope for sensitive strains and highly effective antibiotics. The best outcome for the most favourable balance lies in a multisystem approach to the handling of antibiotics.

The urgent call to curb AMR requires efforts at all levels – governments, healthcare providers, pharmaceutical companies, veterinary and agriculture sectors, and the general public. We can be very optimistic about current deliberate efforts, including the expansion of stewardship in human medicine, animals and agriculture, changing regulations, and creating incentives that promote the discovery of truly novel drugs, as well as ensuring global access to antibiotics. Global society as a collective must share the same perspective of the issues and the vision required to work towards maintaining a strong antimicrobial armament. Antibiotics are a unique biological tool that demands respect, as does any form of Nature. Humans have only recently begun to influence the ecologic balance between antibacterials, bacteria and the resistance mechanisms they display. There is an increasing current understanding that we can favourably restore the balance that preserves and enhances the value of this critical therapeutic class.

## Abbreviations

FDA, Food and drug administration; WHO, World health organization
